# Serotonin 2A (5-HT_2A_) receptor affects cell–matrix adhesion and the formation and maintenance of stress fibers in HEK293 cells

**DOI:** 10.1038/s41598-020-78595-6

**Published:** 2020-12-10

**Authors:** Joe Anand Kumar John Jayakumar, Mitradas. M. Panicker, Basudha Basu

**Affiliations:** 1 Manipal Academy of Higher Education, Manipal, India; 2 National Centre for Biological Sciences, Tata Institute of Fundamental Research, Bangalore, India; 3 Present Address: Department of Physiology and Biophysics, School of Medicine, University of California, Irvine, CA USA; 4 Present Address: Leeds Institute of Medical Research at St. James’s, Faculty of Medicine and Health, Leeds University, Leeds, UK

**Keywords:** Cell culture, Wide-field fluorescence microscopy, Integrins, Cellular neuroscience, Molecular neuroscience

## Abstract

5-HT_2A,_ a G-protein coupled receptor, is widely expressed in the human body, including in the gastrointestinal tract, platelets and the nervous system. It mediates various functions, for e.g. learning, memory, mood regulation, platelet aggregation and vasoconstriction, but its involvement in cell-adhesion remains largely unknown. Here we report a novel role for 5-HT_2A_ in cell–matrix adhesion.

In HEK293 cells, which are loosely adherent, expression and stimulation of human or rat 5-HT_2A_ receptor by agonists such as serotonin or 2,5-dimethoxy-4-iodoamphetamine (DOI) led to a significant increase in adhesion, while inhibition of 5-HT_2A_ by antipsychotics, such as risperidone, olanzapine or chlorpromazine prevented it. 5-HT_2A_ activation gave rise to stress fibers in these cells and was also required for their maintenance. Mechanistically, the 5-HT_2A_-mediated adhesion was mediated by downstream PKC and Rho signaling. Since 5-HT_2A_ is associated with many disorders such as dementia, depression and schizophrenia, its role in cell–matrix adhesion could have implications for neural circuits.

## Introduction

The serotonin receptor subtype, 5-HT_2A_, is a G protein-coupled receptor expressed in multiple regions of the body e.g. gastrointestinal tract, liver, smooth muscles and platelets. It is also expressed in many areas of the central nervous system, which include the cortex and hippocampus^1–3^. It affects behaviour, mood, sleep, thermoregulation and multiple physiological processes such as muscle contraction and platelet aggregation^4–6^. One of the classical roles of 5-HT_2A_ is in platelet-aggregation. Exposure of platelets to vascular injury causes them to release serotonin and aggregate due to activation of their 5-HT_2A_ receptor^7–9^. While implicated in platelet aggregation, which is a form of `cell–cell adhesion’, 5-HT_2A_ is not reported to be involved in ‘cell–matrix adhesion’.

In this study, we demonstrate that either the rat or human 5-HT_2A_ receptors, when stably expressed and activated by agonists^10–13^ in HEK293 cells, caused increased adhesion of these cells to a variety of natural substrates. Conversely, 5-HT_2A_-dependent increase in adhesion was consistently disrupted by antagonists of this receptor, including clinically used antipsychotics. Our experiments were done in DMEM without serum, which was supplemented with ligands, pathway blockers or activators as required.

Dissecting the contributions of a single receptor subtype is difficult in vivo and neural cell lines due to the many serotonin receptor subtypes and multiple receptor subtypes being co-expressed in the same cell. We have limited the contribution of other serotonin receptors in this cell-based system by expressing a single receptor subtype i.e. 5-HT_2A_ in HEK293 cells. This has allowed us not only to establish that the 5-HT_2A_ receptor can play a significant role in affecting cell-substrate adhesion but also allowed us to identify some of the signaling pathways involved.

While the rat and human 5-HT_2A_ share more than 90% sequence homology^14^, they exhibit differences in their functional selectivity, desensitization and resensitization^11,13,15^. Earlier reports from our laboratory had shown differences in internalization of human and rat 5-HT_2A_ when activated by serotonin for e.g. the human 5-HT_2A_ took ~ 6 h to recycle back to cell membrane, whereas the rat 5-HT_2A_ could do so in just 3 h^13,15^. Similarly, human 5-HT_2A_ internalization required β-arrestin and GRK-2 but rat 5-HT_2A_ did not^15,16^. These differences could also be attributed to just a few amino acid differences in the sequences of the two receptors^15^. Our results suggest that though there are differences in signaling between the rat and human receptors, these differences did not extend to cell–matrix adhesion, which was similar between the human and rat 5-HT_2A_ expressed in HEK293 cells. Our current study adds to the functional similarities and differences between human and rat receptors and describes the use of a cellular model which should be of use in the study of human psychiatric disorders like schizophrenia, which are routinely studied using animal model systems.

## Results

### Activation of 5-HT_2A_ leads to adhesion of HEK293 cells to multiple substrates

While HEK293 cells are loosely adherent, expression and stimulation of 5-HT_2A_ leads to an increase in their adhesion to typically used matrices in tissue culture. Our results show that in the absence of 5-HT_2A_ receptor or its stimulation low cell to matrix adhesion and activating the receptor with 10 μM serotonin increased this adhesion significantly. As expected, this increase in adhesion was only seen in the receptor-expressing cells and not in the parental untransfected HEK293 cells, which lack this receptor.

**Figure 1:**
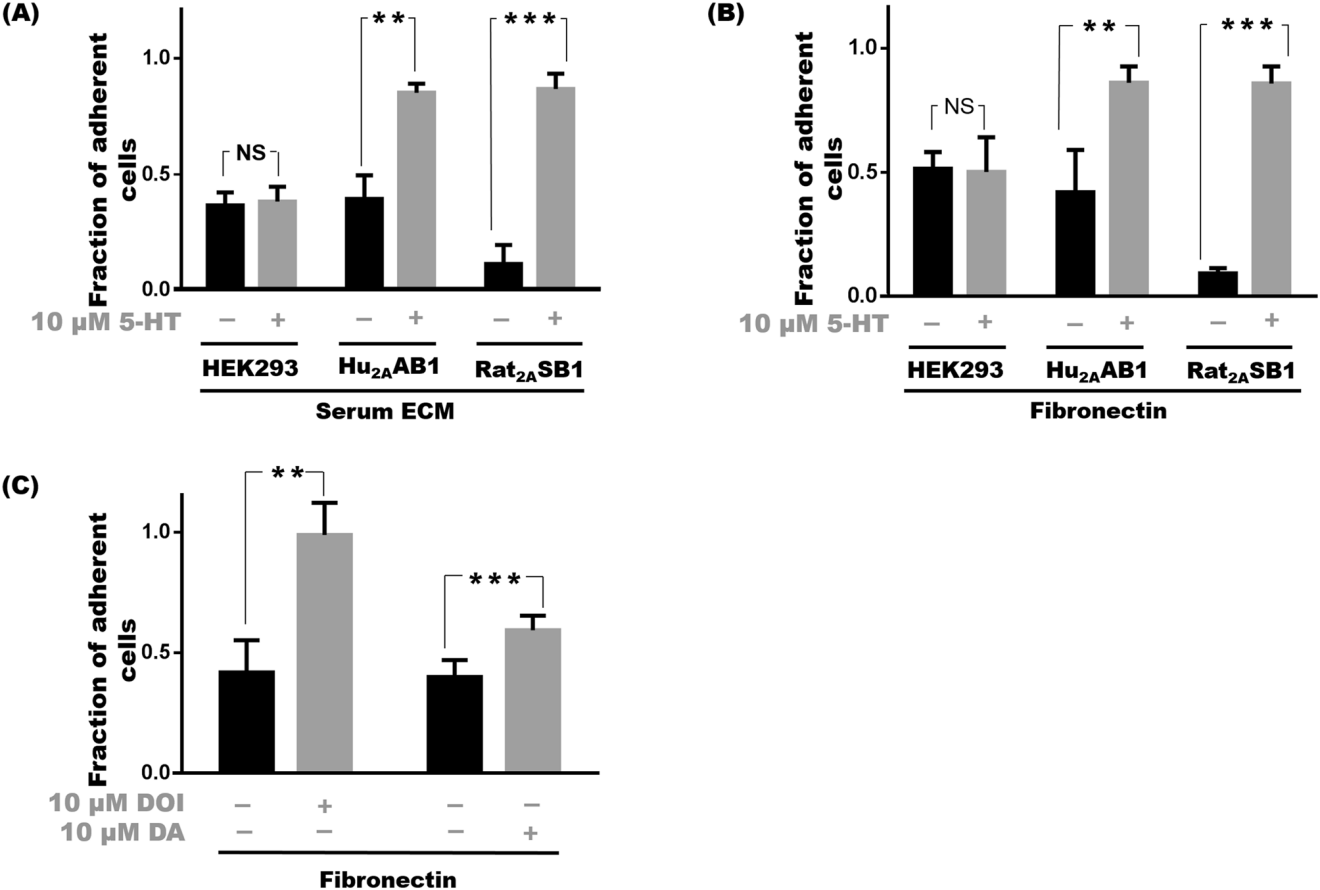
Expression and activation of 5-HT_2A_ leads to adhesion of HEK293 cells to multiple substrates. (**A**) Cell adhesion profile of HEK293 cells, Hu_2A_AB1 and Rat_2A_SB1 cells treated with 10 µM serotonin compared to untreated control on serum ECM substrate. (**B**) Cell adhesion profile of HEK293, Hu_2A_AB1 and Rat_2A_SB1 cells treated with 10 µM 5-HT compared to untreated control on a fibronectin substrate. (**C**) Cell adhesion profile of Hu_2A_AB1 cells on treatment with 10 µM DOI a 5-HT_2A_-specific agonist, and 10 µM dopamine (DA), a partial agonist compared to their respective untreated controls on a fibronectin substrate. Error bars represent SD values. The data represented by the gray bars are compared against the respective black bars by two tailed students t test; p value less than 0.05 is considered significant. The corresponding p value for the asterisks are **p < 0.01, ***p < 0.001, NS p > 0.05.

HEK293 cells exhibited poor adhesion to tissue culture (TC) plastic surfaces pre-treated with DMEM supplemented with 10% fetal bovine serum (FBS) (henceforth referred to as serum ECM^17^), with only 37 ± 6% of cells remaining attached after being subjected to mechanical disruption. Treatment of HEK293 cells with 10 μM serotonin also did not significantly affect adhesion and only 38 ± 6% of HEK293 cells adhered in the presence of serotonin. In the case of Hu_2A_AB1 cells or Rat_2A_SB1 cells i.e. HEK293 cells expressing human or rat 5-HT_2A_ respectively, stimulation with 10 μM serotonin led to increased adhesion under similar conditions. We found 85 ± 4% (p < 0.01) of Hu_2A_AB1 and 87 ± 7% (p < 0.001) of Rat_2A_SB1 cells were adherent in the presence of serotonin, while 39 ± 10% and 11 ± 8% cells of the respective untreated cells, which served as controls, remained adherent (Fig. 1A). Other than the initial treatment of the tissue culture plastic with serum-containing media, all experiments were done in the absence of serum, since serotonin is known to be present in sera. We also did some experiments using dialyzed serum which has negligible serotonin and it did not cause adhesion in the 5-HT_2A_ receptor-expressing cells (data not shown). Earlier studies using Hu_2A_AB1 cells and Rat_2A_SB1 cells^13,15^ had used serotonin at 10 μM concentration to activate 5-HT_2A_. A dose–response study of serotonin-mediated adhesion was carried out on Hu_2A_AB1 cells with serotonin concentrations ranging from 1 nM to 100 μM. Increase in adhesion was observed from 10 nM of serotonin onwards (Supplementary Fig. S2A) and adhesion was seen to be most significant and saturating at 10 μM. We have, therefore, used this concentration as a standard in most of our experiments.

We next examined adhesion on plates pre-coated with fibronectin, a common ECM component and a ligand for several integrins. 5-HT_2A_-mediated adhesion was also observed on this substrate with 86 ± 7% (p < 0.01) of Hu_2A_AB1 cells and 86 ± 7% (p < 0.001) of Rat_2A_SB1 cells adherent at 10 µM serotonin compared to 42 ± 17% for untreated Hu_2A_AB1 and 9 ± 2% for Rat_2A_SB1, which served as controls. The adhesion of HEK293 cells on the plate pre-coated with fibronectin showed no significant difference on treatment with serotonin and 51 ± 5% of untreated HEK293 cells and 49 ± 13% of 10 μM serotonin-treated cells remained adherent (Fig. 1B). We used fibronectin pre-coated TC plastic for most of our subsequent experiments, unless otherwise mentioned, due to its natural occurrence and abundance in various tissues.

Increased adhesion was also seen on stimulation of 5-HT_2A_ with agonists of 5-HT_2A_ e.g. DOI and dopamine. In Hu_2A_AB1 cells, treatment with 10 μM DOI, a synthetic ligand of 5-HT_2A_, caused a massive increase in adhesion with 99 ± 13% of the cells staying adherent compared to only 42 ± 13% of untreated cells (p < 0.01). Treatment with10 μM dopamine, a partial agonist of 5-HT_2A_, also caused a significant increase in the adhesion with 59 ± 6% of cells adherent compared to only 40 ± 7% of untreated cells (p < 0.05) (Fig. 1C). On the other hand, HEK293 cells did not show any increase in the presence of DOI or dopamine (Supplementary Fig. S2B).

We also created a comprehensive dataset compiled from all the experiments done during the entire duration of the study which details the variability in cell-adherence with and without 10 μM serotonin treatment, and is available in Supplementary Fig. S2C,D. The respective datasets for each group were seen to follow a normal distribution as determined by Lilliefors goodness-of-fit test of composite normality at alpha level of 0.05. The result of such a large scale compilation, clearly showed that in the absence of 5-HT_2A_ receptor activation there is lower adhesion, and stimulation of 5-HT_2A_ receptor with 10 μM serotonin led to maximal increases in adhesion (Supplementary Fig. S2C,D). In this dataset, the individual data points in each group did not always belong to the same day, and their respective sample sizes also varied, so we determined the normality of the distribution, and its mean and standard deviation to demonstrate robust reproducibility and consistency in adhesion caused by 5-HT_2A_ expression and activation.

### 5-HT_2A_-mediated adhesion is specific to natural substrates

Since 5-HT_2A_ mediated adhesion was similar on both fibronectin-coated and serum-adsorbed ECM substrates, adhesion was also examined on a non-natural and widely used matrix i.e. poly-d-ornithine used in tissue culture. The adhesion seen with this molecule as substrate is known to be primarily based on electrostatic complementarity^18^, and not dependent on the biological interactions between focal adhesions and ECM components.

**Figure 2:**
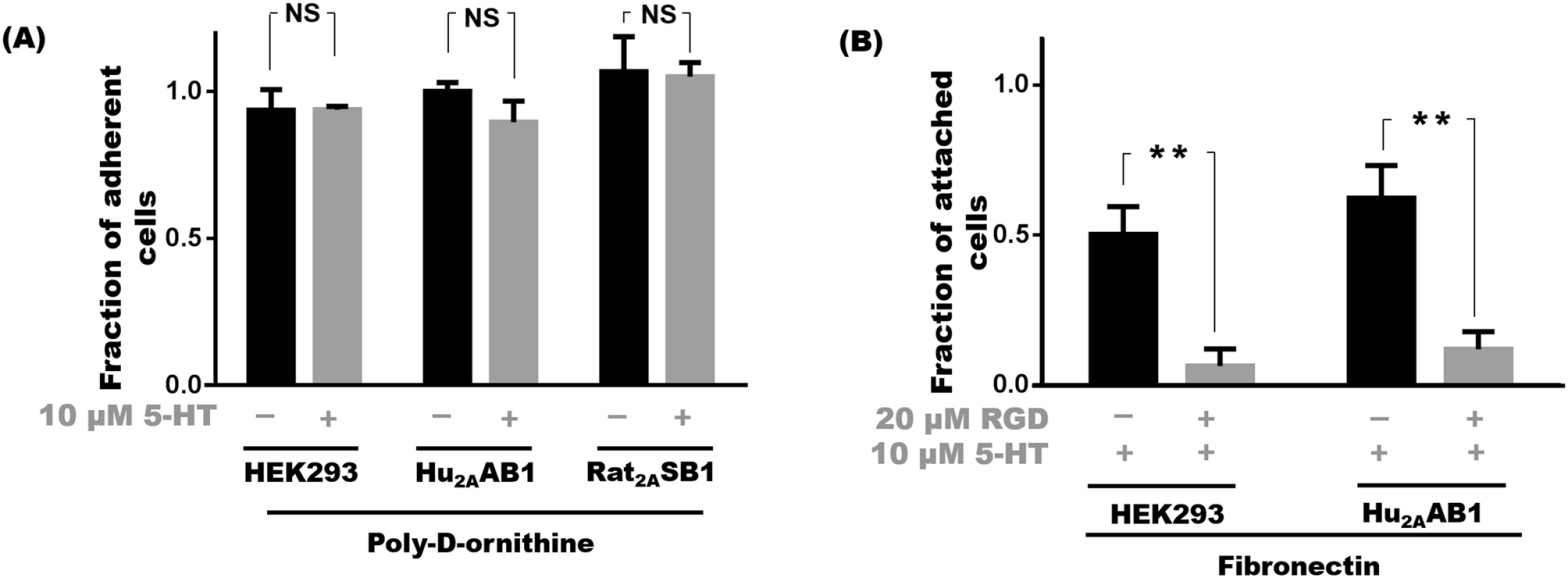
5-HT_2A_-mediated adhesion is specific to natural substrates. (**A**) Cell adhesion profile of HEK293, Hu_2A_AB1 and Rat_2A_SB1 cells on poly-d-ornithine substrates with 10 µM 5-HT compared to untreated controls. (**B**) Cell attachment profile of Hu_2A_AB1 and HEK293 cell suspensions with or without pre-treatment with10 µM RGD peptide compared to non pre-treated controls. Error bars represent SD values. The data represented by the gray bars are compared against the respective black bars by two tailed students t test; p value less than 0.05 is considered significant. The corresponding p value for the asterisks are **p < 0.01, NS p > 0.05.

In previously examined substrates, Hu_2A_AB1 or Rat_2A_SB1 cells showed an increase in adhesion only when 5-HT_2A_ was activated by an agonist; but on the synthetic substrate poly-d-ornithine, Hu_2A_AB1 and Rat_2A_SB1 cells remained fully adherent in the absence of any ligand. On poly-d-ornithine, 100 ± 3% of Hu_2A_AB1 and 107 ± 12% Rat_2A_SB1 cells were adherent in the absence of serotonin compared to 90 ± 7% and 105 ± 5% in the presence of 10 µM serotonin respectively (Fig. 2A). Similarly, HEK293 cells which lack the 5-HT_2A_ receptor, showed poor adhesion on all natural substrates irrespective of the presence of serotonin. On poly-d-ornithine however, they exhibited strong non-specific adhesion with 94 ± 4% untreated and 94 ± 1% of 10 μM serotonin-treated cells adhering (Fig. 2A). It appears that all three cell lines attach well on poly-d-ornithine which causes adhesion via electrostatic interactions, and the adhesion is independent of 5-HT_2A_ activity. Hence, we conclude that 5-HT_2A_-mediated adhesion is observed only on biologically relevant matrices and that 5-HT_2A_ activity not essential for cell adhesion on a non-natural substrate like poly-d-ornithine.

As 5-HT_2A_-mediated adhesion was found to be relevant only to natural substrates, but not with an artificial substrate such as poly-d-ornithine, we examined the biological processes involved. Since integrins engage with the RGD motifs of various ECM proteins including fibronectin, interfering with integrin–fibronectin interactions using RGD peptides would be expected to decrease cell attachment. For investigating the role of integrins in the attachment processes in these cells, cell-attachment was assayed in the presence and absence of RGD peptides. Briefly, enzyme-free cell dissociation buffer was used to dislodge the cells and cell suspension was exposed to RGD peptides prior to plating onto fibronectin-coated wells. The number of cells attached over a period of time was determined using PrestoBlue dye. We found that a 60-min pre-treatment with 20 μM RGD peptide, reduced the attachment of Hu_2A_AB1 to12 ± 6%, whereas in the absence of RGD peptide, 62 ± 11% of cells attached over a period of 60 min (p < 0.005). Similarly, only 6 ± 6% of HEK293 cells pre-treated with 20 μM RGD peptide attached, compared to 50 ± 9% of cells that attached in the absence of RGD peptides (Fig. 2B) (p < 0.005) showing an involvement of integrins in this process.

This phenomenon of 5-HT_2A_-mediated cell–matrix adhesion is distinct from any effects of serotonylation of fibronectin^19^. To rule out contributions from this process, we examined adhesion in the presence of cystamine, an inhibitor of transglutaminase, which blocks serotonylation. We found that on a fibronectin matrix, cystamine had no significant effect on 5-HT_2A-_mediated adhesion (Supplementary Fig. S2E).

### Antipsychotics cause decrease in 5-HT_2A_-mediated adhesion

Since stimulation of 5-HT_2A_ by agonists in Hu_2A_AB1 and Rat_2A_SB1 cells caused an increase in the adhesion, the effect of antagonists/inverse agonists of 5-HT_2A_, some of which are antipsychotic drugs_,_ was probed. One of the characteristics of antipsychotics, especially atypical ones like risperidone, is their high affinity for 5-HT_2A_. As expected, various antipsychotics blocked agonist-stimulated increase in 5-HT_2A_-mediated adhesion. The loss of adhesion seen on inhibition of 5-HT_2A_ was applicable only to natural substrates and not to poly-d-ornithine.

**Figure 3:**
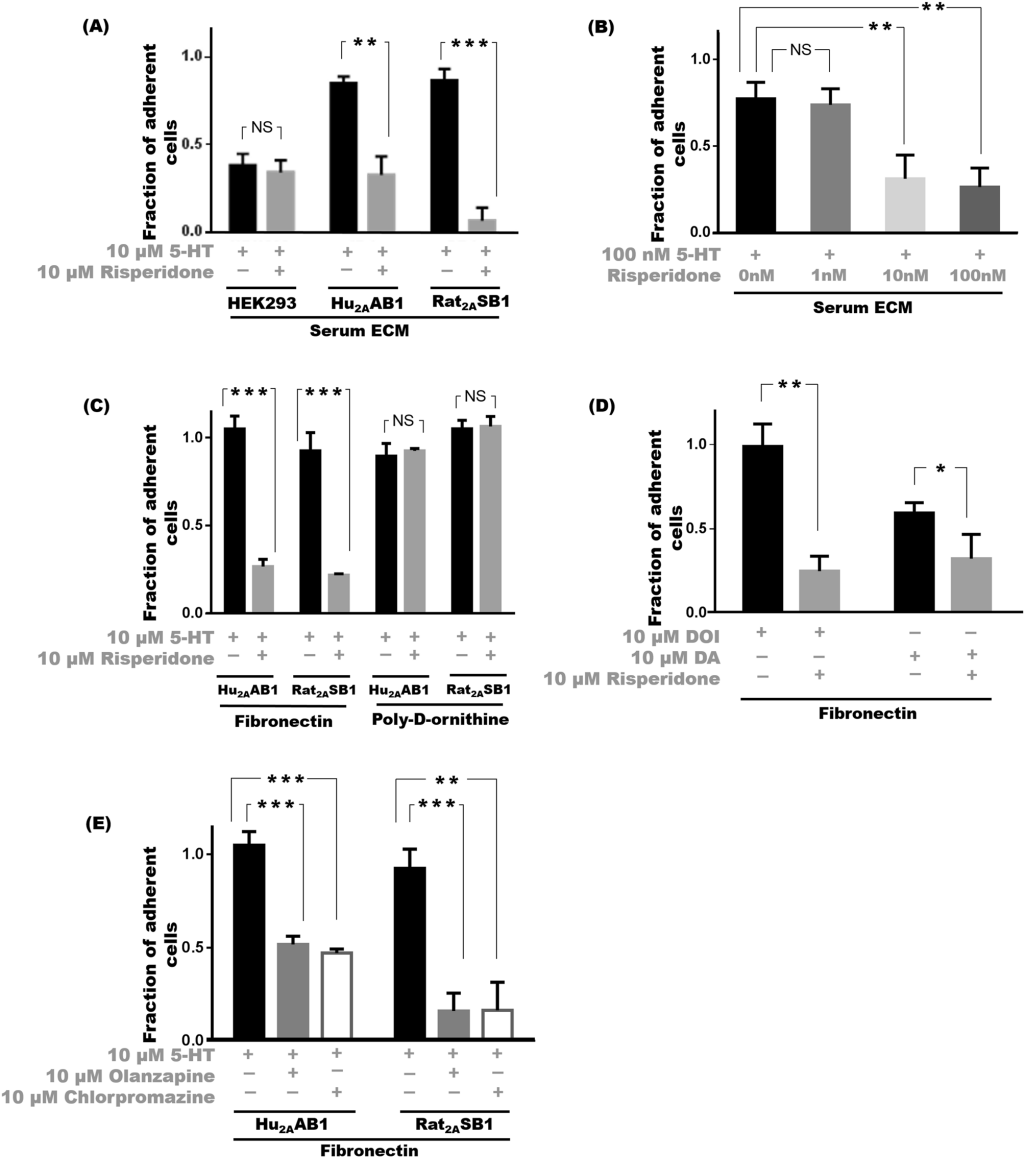
Inhibition of 5-HT_2A_ by antipsychotics leads to loss of adhesion. (**A**) Cell adhesion profile of HEK293 cells, Hu_2A_AB1 or Rat_2A_SB1 cells treated with 10 µM risperidone and 10 µM 5-HT compared to 10 µM 5-HT treatment alone on serum ECM substrate. (**B**) Cell adhesion profile of Hu_2A_AB1 cells treated with varying concentration of risperidone—1 nM, 10 nM or 100 nM, in the presence of 100 nM serotonin compared to 100 nM 5-HT treatment alone on a serum ECM substrate. (**C**) Cell adhesion profile of HEK293, Hu_2A_AB1 and Rat_2A_SB1 cells in the presence of 10 µM risperidone and 10 µM 5-HT compared to control cells treated only with 10 µM 5-HT treatment on fibronectin or poly-d-ornithine substrates. (**D**) Cell adhesion profile of Hu_2A_AB1 cells treated with 10 μM risperidone along with either 10 μM DOI or 10 μM dopamine (DA) compared to their respective controls without risperidone on a fibronectin substrate. (**E**) Cell adhesion profile of Hu_2A_AB1 and Rat_2A_SB1 cells with 10 μM olanzapine or 10 μM chlorpromazine and 10 µM 5-HT treatments compared to 10 µM 5-HT treatment alone, on fibronectin substrate. Error bars represent SD values. The data represented by the gray bars are compared against the respective black bars by two tailed students t test; p value less than 0.05 is considered significant. The corresponding p value for the asterisks are *p < 0.05, **p < 0.01, ***p < 0.001, NS p > 0.05.

Inhibition of 5-HT_2A_ by risperidone prevented serotonin-mediated increase in adhesion of Hu_2A_AB1 and Rat_2A_SB1 cells on serum-ECM coated plates. On stimulation of 5-HT_2A_ receptor with 10 μM 5-HT, 85 ± 4% of Hu_2A_AB1 cells and 87 ± 7% of Rat_2A_SB1 remained adherent; but when 10 μM risperidone was also present the adhesion decreased, with only 33 ± 11% (p < 0.01) of Hu_2A_AB1 and 7 ± 7% Rat_2A_SB1 (p < 0.0005) adherent respectively. But in the case of HEK293 cells, just like serotonin, risperidone was found not to affect adhesion and 38 ± 6% of 10 μM serotonin-treated cells remained adherent as compared to 34 ± 7% cells treated with both 10 μM risperidone and 10 μM serotonin (Fig. 3A).

Since we observed that the increased adhesion seen with 10 μM serotonin was abolished by 10 μM risperidone, we examined the effect of decreasing doses of risperidone on adhesion mediated by a lower concentration of serotonin. A dose–response study on Hu_2A_AB1 cells in the presence of 100 nM serotonin and varying concentrations of risperidone (1 nM, 10 nM and 100 nM) showed that while 100 nM serotonin caused high levels of adhesion with 77 ± 10% cells adherent, risperidone at 10 nM completely abolished it. This was indistinguishable from results obtained with 100 nM risperidone treatment i.e. 31 ± 14% (p < 0.01) and 26 ± 11% (p < 0.005) cells remaining adherent in the presence 10 nM and 100 nM risperidone. 1 nM risperidone did not cause any significant reduction and 74 ± 9% cells remained adherent (p > 0.05) (Fig. 3B). The doses of risperidone needed to block 5-HT_2A_-mediated adhesion were also in accordance with the known affinity of risperidone for the 5-HT_2A_ receptor.

Risperidone affected 5-HT_2A_-mediated adhesion, on plates pre-coated with fibronectin as well. While 10 μM serotonin significantly increased adhesion of Hu_2A_AB1 cells and Rat_2A_SB1 cells with 105 ± 7% and 93 ± 10% respectively becoming adherent, this decreased significantly in the presence of 10 μM risperidone with only 27 ± 4% (p < 0.001) of Hu_2A_AB1 and 22 ± 1% (p < 0.0005) of Rat_2A_SB1 cells remaining adherent. However, on poly-d-ornithine, inhibition of the receptor had no effect, and treatment with risperidone did not result in any decrease in adhesion with 90 ± 7% of 10 µM serotonin-treated Hu_2A_AB1 cells remaining adherent compared to 93 ± 1% of cells when 10 μM risperidone was also present. Similarly, Rat_2A_SB1 cells did not exhibit any decrease in adhesion on poly-d-ornithine substrates when the 5-HT_2A_ antagonist was present. In the presence of 10 µM serotonin alone 105 ± 5% of cells was adherent and when 10 µM risperidone was also present a comparable 106 ± 6% of cells was found to be adherent (Fig. 3C).

This observed loss of adhesion on inhibition of 5-HT_2A_ by antipsychotics was also determined with other 5-HT_2A_ agonists besides serotonin. All the antipsychotics we tested blocked the increase in adhesion triggered by complete and partial agonists of the 5-HT_2A_. Risperidone abolished the increase in adhesion seen with activation of 5-HT_2A_ by DOI, a specific agonist, and dopamine, a partial agonist. In Hu_2A_AB1 cells, 10 µM DOI caused 99 ± 13% of cells to adhere as compared to 25 ± 9% of cells when 10 µM risperidone was also present (p < 0.005). Likewise, in the presence of the partial agonist dopamine at 10 µM, 59 ± 6% of cells were adherent which decreased to 32 ± 14% when 10 µM risperidone was also present (p < 0.05) (Fig. 3D). Loss of adhesion due to inhibition of 5-HT_2A_ was also consistently seen with other antipsychotics such as olanzapine, an atypical antipsychotic, and chlorpromazine, a typical antipsychotic. In Hu_2A_AB1 cells, 10 μM serotonin treatment resulted in 105 ± 7% cells being adherent, but when either 10 μM olanzapine or 10 μM chlorpromazine was also present in addition to serotonin, adhesion decreased significantly and only 57 ± 4% (p < 0.0005) and 47 ± 2% (p < 0.0005) cells remained adherent respectively. Similarly 93 ± 10% of Rat_2A_SB1 cells were adherent with 10 μM serotonin treatment, but the adhesion was significantly reduced when 10 μM olanzapine or 10 μM chlorpromazine was also present, with only 11 ± 1% (p < 0.0005) and 6 ± 3% (p < 0.005) cells remaining adherent (Fig. 3E).

### Stress fibers are modulated by the activity of 5-HT_2A_

Since cell-substrate adhesion is associated with stress fibers, their role was examined in 5-HT_2A_-mediated adhesion. Phalloidin Alexa 568, which binds to F-actin was used to detect stress fibers. Stress fibers were seen to be formed or disrupted based on 5-HT_2A_ activity, in the cells examined. Stimulation of 5-HT_2A_ was found to be essential for the formation and maintenance of stress fibers and conversely inhibition of 5-HT_2A_ resulted in the disruption of stress fibers.

**Figure 4:**
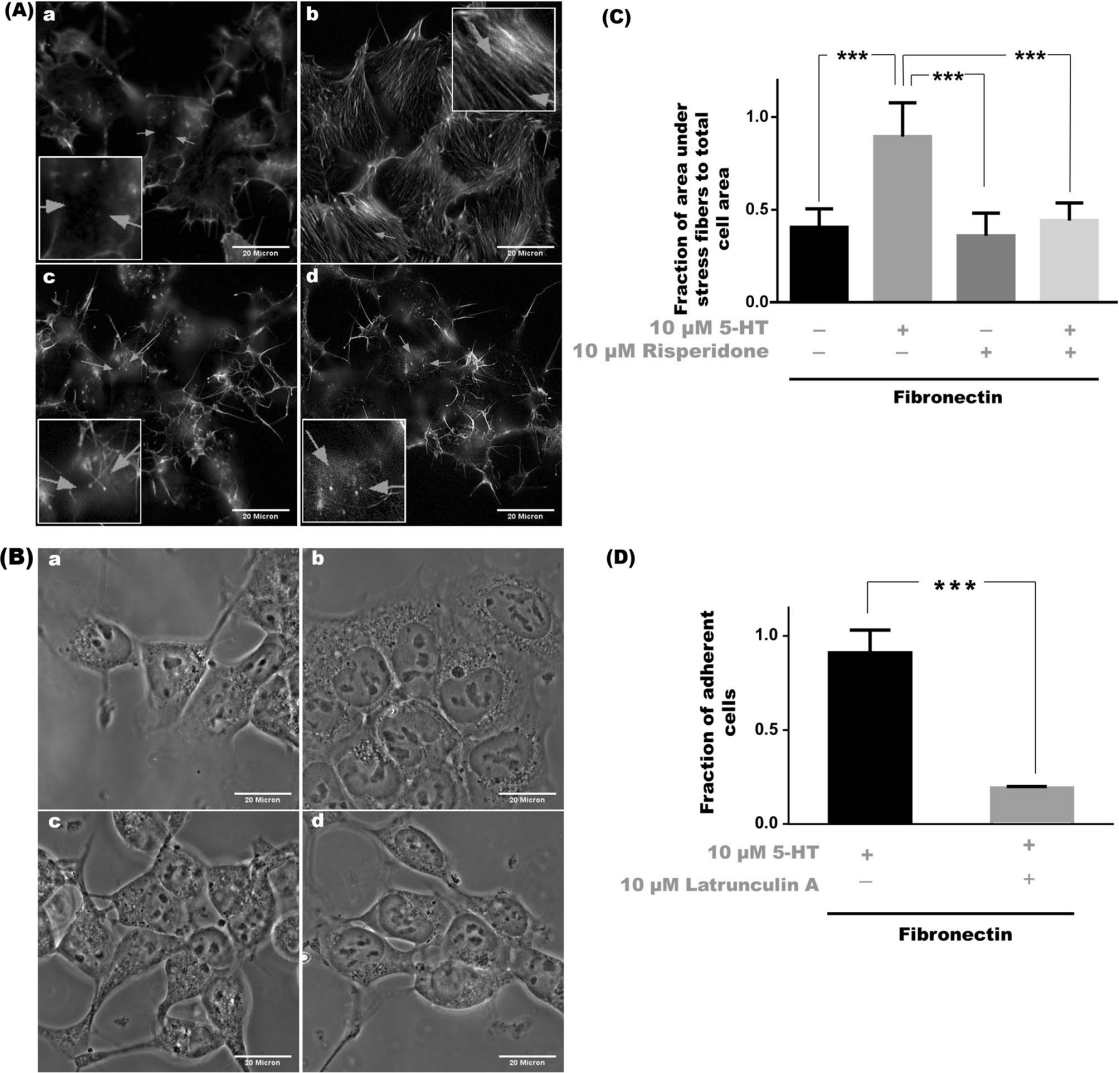
Stress fibers are modulated by the activity of 5-HT_2A_ in Hu_2A_AB1 cells. (**A**) Stress fibers appear to be absent in untreated control cells (**a**) and appear on treatment with 10 μM 5-HT (**b**), they are no longer visible on treatment with 10 μM risperidone alone (**c**) or on treatment with 10 μM risperidone + 10 μM 5-HT (**d**). Stress fibers stained with Phalloidin Alexa 568 are seen as long structures indicated by the arrows. Scale bar represents 20 µm for all images. (**B**) The corresponding phase contrast images of cells stained with Phalloidin Alexa 568 in (**A**) are shown in (**a**), (**b**–**d**). Scale bar represents 20 µm for all images. (**C**) The fraction of area occupied by stress fibers by the total area occupied by cells with 10 μM 5-HT, 10 μM risperidone and a combination of 10 μM risperidone + 10 μM 5-HT treatment compared to untreated control. (**D**) Cell adhesion profile of Hu_2A_AB1 cells treated with 10 µM 5-HT in the presence or absence of 10 µM latrunculin A on a fibronectin substrate. Error bars represent SD values. The data represented by the gray bars are compared against the respective black bars by two tailed students t test; p value less than 0.05 is considered significant. The corresponding p value for the asterisks is ***p < 0.001.

In untreated Hu_2A_AB1 cells without 5-HT_2A_ stimulation, stress fibers were absent and stimulation of 5-HT_2A_ by 10 μM serotonin resulted in robust stress fiber formation. Conversely, inhibition of 5-HT_2A_ by 10 μM risperidone, resulted in the disruption of stress fibers; and the fibers remained disrupted even in the presence of 10 μM serotonin (Fig. 4A). The phase contrast images of the Phalloidin stained cells are presented in Fig. 4B.

The area occupied by stress fibers in a cell increased with 5-HT_2A_ stimulation and decreased with inhibition, a trend similar to adhesion. In untreated control conditions, 40 ± 10% of total area occupied by cells had stress fibers, but in cells treated with10 μM serotonin a significant increase with 89 ± 18% (p < 0.001) of cell area having stress fibers was observed. Treatment with 10 μM risperidone alone or in the presence of 10 μM serotonin reduced stress fiber formation with 44 ± 10% (p < 0.001) area with risperidone alone and 36 ± 12% (p < 0.001) on co-treatment with serotonin (Fig. 4C). However, it is important to note that although there was no significant difference in the total cell area between control and serotonin-treated groups, the area occupied by a cell is significantly reduced when treated with risperidone as the cell begins to lose adhesion and round up (Supplementary Fig. S3A,B).

Since, stimulation of 5-HT_2A_ led to the formation of stress fibers and increased adhesion, the effect of disruption of stress fibers on adhesion was explored. Stress fibers were therefore disrupted using latrunculin A under conditions of 5-HT_2A_-mediated adhesion. In Hu_2A_AB1 cells, treatment with 10 μM serotonin alone caused 91 ± 11% of cells to remain adherent but when 10 μM latrunculin A was used to disrupt stress fibers, only 19 ± 1% (p < 0.001) of the cells remained adherent (Fig. 4D).

### PKC and Rho GTPase are involved in 5-HT_2A_-mediated adhesion

Since stimulation of 5-HT_2A_ in HEK293 cells results in increased adhesion along with stress fiber formation; the events downstream of 5-HT_2A_ stimulation, namely Protein Kinase C (PKC) activation and intracellular Ca^2+^ rise, and the signaling molecules associated with adhesion and stress fiber formation i.e. PKC and Rho GTPases, were probed.

**Figure 5:**
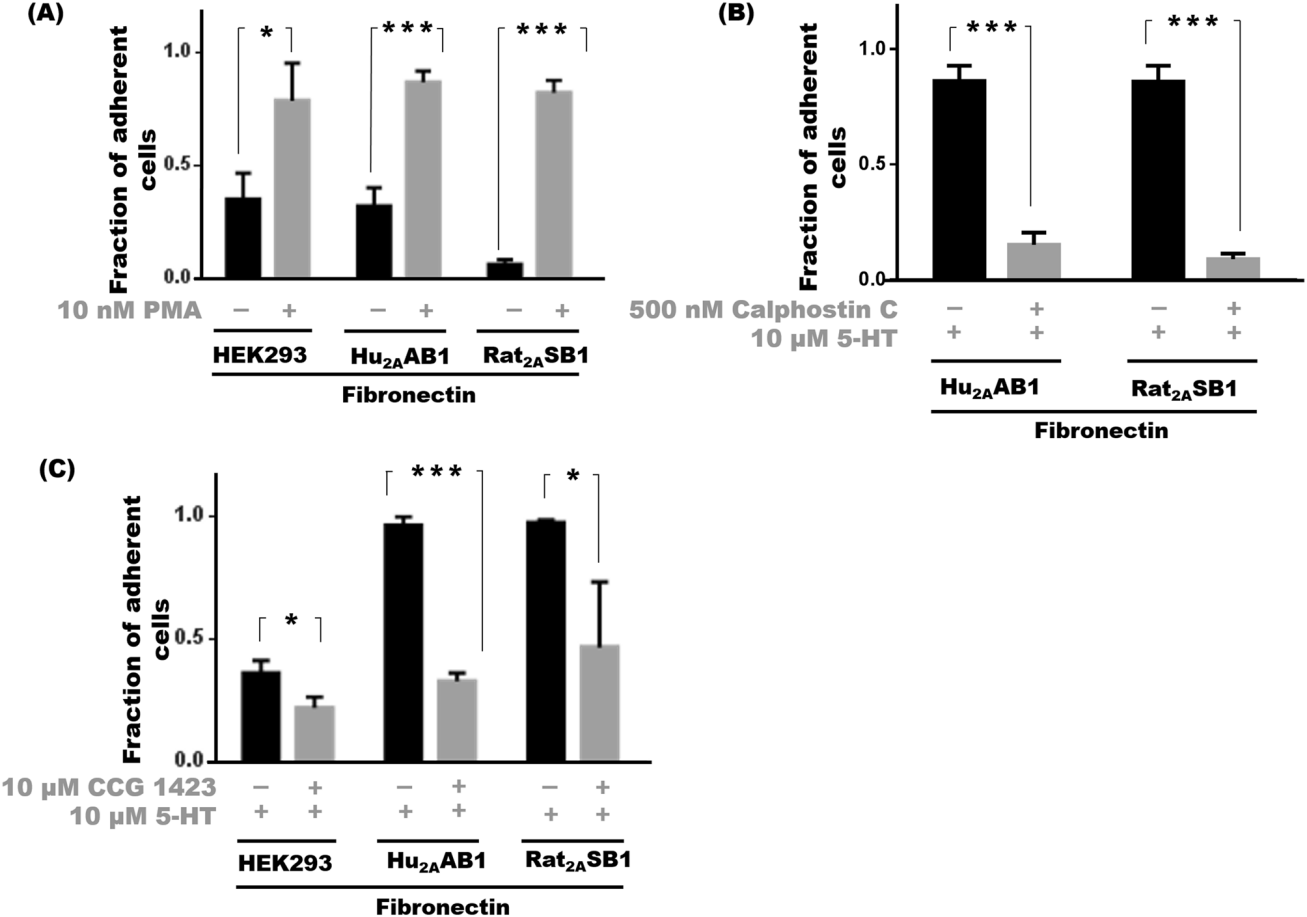
PKC and Rho GTPase are involved in 5-HT_2A_-mediated adhesion. (**A**) Cell adhesion profile of HEK293, Hu_2A_AB1 and Rat_2A_SB1 cells treated with 10 nM PMA compared to untreated controls on fibronectin substrate. (**B**) Cell adhesion profile of HEK293, Hu_2A_AB1 and Rat_2A_SB1 cells treated with 10 μM 5-HT with either no pre-treatment or with pre-treatment with 500 nM light-activated calphostin C on a fibronectin substrate. (**C**) Cell adhesion profile of HEK293, Hu_2A_AB1 and Rat_2A_SB1 cells after a 24-h treatment with 10 μM CCG-1423 + 10 μM 5-HT compared to 10 μM 5-HT treatment alone on a fibronectin substrate. Error bars represent SD values. The data represented by the gray bars are compared against the respective black bars by two tailed students t test; p value less than 0.05 is considered significant. The corresponding p value for the asterisks are *p < 0.05, **P < 0.01, ***p < 0.001, NS p > 0.05.

In Hu_2A_AB1 and Rat_2A_SB1 cells, PKC activation by 10 nM PMA alone caused an increase in adhesion with 87 ± 5% (p < 0.001) and 82 ± 5% (p < 0.001) of cells being adherent respectively while in the corresponding vehicle-treated controls, only 32 ± 8% of Hu_2A_AB1 cells and 6 ± 2% of Rat_2A_SB1 cells were adherent. HEK293 cells also exhibited increased adhesion with 10 nM PMA and 79 ± 16% of treated cells were adherent compared to untreated control cells where only 35 ± 11% of the cells were adherent (p < 0.05) (Fig. 5A).

Consistent with this, stimulation of 5-HT_2A_ by serotonin did not lead to increased adhesion in Hu_2A_AB1 and Rat_2A_SB1 cells when activation of PKC, was blocked by light-activated calphostin C. While treatment with 10 μM serotonin treatment caused 86 ± 7% of Hu_2A_AB1 cells and 86 ± 7% cells of Rat_2A_SB1 cells to remain adherent; the presence of 500 nM light-activated calphostin C along with serotonin decreased adhesion to 15 ± 3% for Hu_2A_AB1cells (p < 0.001) and 9 ± 2% for Rat_2A_SB1 (p < 0.001) cells (Fig. 5B). HEK293 cells also showed a slight decrease in adhesion with 500 nM calphostin C treatment alone with 24 ± 1% of cells adherent compared to 39 ± 2% of untreated control cells (p < 0.001). Similarly, Hu_2A_AB1 cells showed decreased adhesion with calphostin C treatment, with 42 ± 17% control cells and only 12 ± 5% of 500 nM calphostin C treated cells staying adherent (p < 0.05). Rat_2A_SB1 cells on the other hand were poorly adherent to begin with and did not show decreased adhesion with calphostin C treatment alone with 7 ± 3% of 500 nM calphostin C treated and 9 ± 2% control cells remaining adherent (Supplementary Fig. S4A).

We then examined the role of Rho in serotonin-mediated adhesion of Hu_2A_AB1 and Rat_2A_SB1 cells. Following a 24 h treatment with 10 μM serotonin using a previously published protocol^20^, we found that 96 ± 3% of Hu_2A_AB1 cells and 97 ± 1% of Rat_2A_SB1 cells were adherent; but the same treatment coupled with 10 μM CCG-1423, a Rho signaling blocker, resulted in decreased adhesion, with only 33 ± 3% of Hu_2A_AB1 (p < 0.001) and 47 ± 27% of Rat_2A_SB1 (p < 0.05) remaining adherent. In HEK293 cells, treatment with 10 μM CCG-1423 for 24 h significantly decreased adhesion compared to serotonin-treated control cells also treated for the same time period, where only 24 ± 3% of 10 μM CCG-1423 were adherent compared to 37 ± 3% of control cells (p < 0.01) (Fig. 5C). From the above results, we conclude that blocking Rho signaling with CCG-1423 had a far greater effect on serotonin-mediated adhesion.

### Intracellular Ca^2+^ plays a species-specific role in rat and human 5-HT_2A_ mediated adhesion

**Figure 6:**
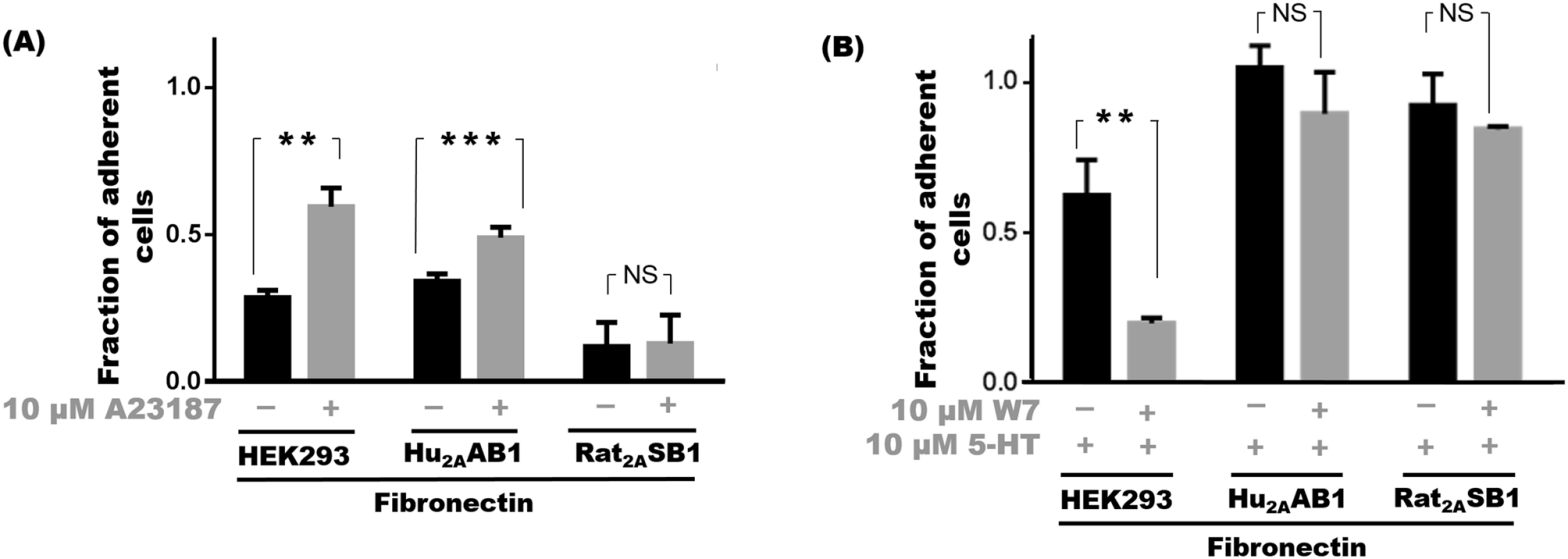
Differential involvement of intracellular calcium increase in 5-HT_2A_ mediated adhesion. (**A**) Cell adhesion profile of HEK293, Hu_2A_AB1 and Rat_2A_SB1 cells treated with 2 μM A23187 treatment compared to untreated controls on fibronectin substrate. (**B**) Cell adhesion profile of HEK293, Hu_2A_AB1 and Rat_2A_SB1 cells treated with 10 µM 5-HT with or without pre-treatment with 10 µM W7 on a fibronectin substrate. Error bars represent SD values. The data represented by the gray bars are compared against the respective black bars by two tailed students t test; p value less than 0.05 is considered significant. The corresponding p value for the asterisks are **p < 0.01, ***p < 0.001, NS p > 0.05.

To analyse if the intracellular Ca^2+^ rise that follows 5-HT_2A_ stimulation plays a role in adhesion, a calcium ionophore, A23187, was used to increase cytoplasmic Ca^2+^. A significant increase in adhesion was seen in the case of HEK293 cells and Hu_2A_AB1 cells with A23187. Increasing cytoplasmic Ca^2+^ with 2 μM A23187 treatment caused a substantial increase in adhesion of HEK293 cells with 60 ± 6% of the cells being adherent compared to 29 ± 2% of untreated control cells (p < 0.01). Similarly, in Hu_2A_AB1 cells, increasing cytoplasmic Ca^2+^ with 2 μM A23187 resulted in a significant increase in adhesion with 49 ± 3% cells adherent compared to 34 ± 2% for control cells (p < 0.001); however, in Rat_2A_SB1 cells the adhesion was unaffected by 2 μM A23187 and was indistinguishable from the control cells with only 12 ± 8% and 13 ± 10% cells remaining adherent respectively (p > 0.05) (Fig. 6A).

As cytoplasmic Ca^2+^ rise causes activation of many pathways including calmodulin pathways, W7, a pharmacological blocker of calmodulin, was used to probe its involvement. We found that W7 did not affect serotonin-mediated adhesion in Hu_2A_AB1 and Rat_2A_SB1 cells. While 10 μM serotonin alone caused 105 ± 7% and 93 ± 10% of cells to adhere respectively in the case of Hu_2A_AB1 and Rat_2A_SB1 cells, the presence of 10 μM W7 had negligible effect and 90 ± 8% of Hu_2A_AB1 cells and 84 ± 3% of Rat_2A_SB1 cells remained adherent. In contrast, in HEK293 cells, adhesion was reduced by W7 irrespective of the presence or absence of serotonin (Fig. 6B, Supplementary Fig. S4B). We found that a combination of 10 μM W7 and 10 μM serotonin treatment reduced cell-adherence to 20 ± 6% compared to cells treated with 10 μM serotonin alone which were 63 ± 12% (p < 0.01) adherent (Fig. 6B). In the absence of serotonin only 20 ± 2% of the 10 μM W7 treated HEK293 cells remained adherent compared to 56 ± 12% of untreated control (p < 0.001) as well (Supplementary Fig. S4B). Interestingly, we also observed that Hu_2A_AB1and Rat_2A_SB1 cells responded differently to W7 alone. In the case of Hu_2A_AB1 cells, there was decrease in adhesion when cells were treated with 10 μM W7 alone and only 20 ± 2% of remained adherent compared to 35 ± 6% of untreated control cells (p < 0.05). But in Rat_2A_SB1 cells, treatment with W7 showed no effect on adhesion with 24 ± 1% of 10 μM W7 cells and 24 ± 1% untreated control cells remaining adherent (Supplementary Fig. S4B).

## Discussion

5-HT_2A_ is a widely studied serotonin receptor, largely owing to its suspected role in neuropsychiatric disorders such as schizophrenia and for being a target of antipsychotic drugs and hallucinogens^21–23^. In particular many antagonists/inverse agonists in schizophrenia for e.g. atypical antipsychotics are believed to act predominantly through this receptor^24,25^. In our earlier studies, 5-HT_2A_ agonists such as serotonin and DOI, or dopamine—a partial agonist were shown to activate the receptor and cause an intracellular Ca^2+^ rise in both Rat_2A_SB1 and Hu_2A_AB1 cells^11–13,15^. We report here that activation of 5-HT_2A_ by these ligands also results in increased adhesion of Rat_2A_SB1 and Hu_2A_AB1 cells to ‘natural’ substrates such as fibronectin and seems to be mediated through integrins. Since untransfected HEK293 cells do not express 5-HT_2A_^13,26^, serotonin does not affect the adhesion of the parental cell line to these substrates. In the present study, risperidone, olanzapine and chlorpromazine were also seen to inhibit 5-HT_2A_ mediated adhesion, and treatment with serotonin, DOI or dopamine increased adhesion via activation of the 5-HT_2A_ though they were unable to overcome antagonist-mediated loss of adhesion (Supplementary Fig. S5A). Atypical antipsychotics such as risperidone^27^ have higher affinities for the 5-HT_2A_ receptor than serotonin, DOI or dopamine and can therefore likely to prevent its activation. This could explain why antipsychotics were able to cause a decrease in adhesion even in the presence of serotonin and other agonists.

In our study we also found that 5-HT_2A_ affected adhesion to natural ECM components such as serum ECM adsorbed onto tissue culture plastic or plates pre-coated with fibronectin, but not to poly-d-ornithine, a ‘non-natural’ substrate. Integrins, the cell adhesion molecules that interact with fibronectin^28–30^, were also seen to be involved in 5-HT_2A_-mediated adhesion. In case of poly-d-ornithine which has multiple positively charged amino groups, the adhesion observed is not integrin-dependent and is instead based on enhancing electrostatic interactions between the coated surface and the negatively charged residues in the cell membrane^18^. This interaction seems to override 5-HT_2A_-mediated adhesion in HEK293-based cell lines.

While we have done our experiments in serum-free media since serum contains serotonin, is known that many cells require FBS for attachment, flattening and growth in vitro^17^. We found that Hu_2A_AB1 and Rat_2A_SB1 cells attached strongly in serum-containing media while HEK293 cells always exhibited poor adhesion. As our work suggests, the serotonin in the serum can cause such an increase in adhesion in the Hu_2A_AB1 and Rat_2A_SB1 cells, while HEK293 cells without the 5-HT_2A_ receptor fail to adhere as strongly. Serum also provides many other ECM components or factors for cell attachment and spreading, which might not involve 5-HT_2A_ directly.

We found that in the absence of other pre-coating, the adsorption of ECM components from serum-containing media onto tissue-culture plastic was essential for the cell lines to adhere and we used this for our initial experiment. Once the cells adhere to these adsorbed matrices, they flatten and secrete their own ECM components over time^31^. It must be noted that our cells do not adhere strongly onto TC plastic in the absence of FBS, but begin to do so in media supplemented with FBS, as early as 10–15 min. This is consistent with the sequence and expected timescale of the events involved in cell-adhesion for e.g. adsorption of ECM components from the serum onto to TC plastic to form a substrate onto which cells attach^31,32^. In our experiments, we used fibronectin as the substrate, because we found adhesion of the cell lines we used to be primarily driven by cell adhesion molecules complementary to RGD peptides.

On stimulation, 5-HT_2A,_ like most G-protein coupled receptors (GPCRs), is known to activate several signaling pathways^33^, and there are reports that some of the conventionally associated pathways such as PKC are also linked with adhesion, e.g. some subtypes of PKC have roles in adhesion and migration in certain cell types^34–36^. Similarly, the Rho family of small GTPases are known to be associated in actin cytoskeleton dynamics and regulate gene-expression associated with adhesion-related processes^37^. GPCRs like the 5-HT_2A_ are known to activate Rho GTPases—RhoA, Rac and cdc42^37–39^. In our study therefore, PKC and Rho GTPases were examined for their roles in 5-HT_2A_-mediated adhesion. We found that they do indeed play significant roles in the 5-HT_2A_-mediated adhesion.

Intracellular Ca^2+^ signaling, is known to play very important roles in cell adhesion and flattening^40,41^. However, in our study, an increase of intracellular calcium resulted in an increase in adhesion only in Hu_2A_AB1 and not Rat_2A_SB1 cells, which could be due to differences between human and rat receptors, and curiously blocking calmodulin did not cause a decrease in adhesion in either cell line.

In the present study, PKC is seen to be indispensable for 5-HT_2A_-mediated adhesion, which is not surprising as it is a part of the canonical 5-HT_2A_ signaling pathway and is commonly associated with adhesion. We see increased adhesion with PKC activation and a loss of adhesion on blocking it even in the presence of serotonin, emphasizing the central role played by PKC in this process. If PKC is rendered inactive, stimulation of 5-HT_2A_ is no longer capable of causing an increase in adhesion. The fact that untransfected HEK293 cells also show an increased adhesion with PKC activation and reduced adhesion with PKC inactivation suggests that the molecular players that the 5-HT_2A_ receptor uses for mediating adhesion is also present in HEK293 but stays inactive since it cannot be activated by serotonin in the absence of the 5-HT_2A_ receptor.

Rat_2A_SB1 cells did not show any increase in adhesion with calcium ionophore A23187 treatment, although Hu_2A_B1and HEK293 cells and cells did show a moderate increase. However, blocking the calmodulin pathways by W7 did not result in any loss of 5-HT_2A_-mediated adhesion. Taken together, the results suggest there are significant species-specific differences in the roles for calcium signaling in 5-HT_2A_ mediated adhesion and that Calmodulin may not play a role. Therefore, we conclude that, barring species-specific differences, the stimulation of 5-HT_2A_ results has a robust effect on adhesion, with a comparable increase also seen on PKC activation by PMA that suggests a key role for PKC. The pathways in play are summarized in Supplementary Fig. S5B.

In *Drosophila,* knocking out 5-HT_2Dro_ (the fly 5-HT_2A_ ortholog) causes aberrant gastrulation and altered sub-cellular localization of adherens junction and lethality in developing embryos^42,43^. In mammals, although serotonin^44–47^ and 5-HT_2A_^48^ are expressed early in development, their roles do not seem to be indispensable, for e.g. 5-HT_2A_-deficient mice are viable, fertile and grossly normal, which might be due to compensation by the remaining two 5-HT_2_ receptor subtypes which have similar downstream signaling. However, all ex utero cultured mouse embryos treated with atypical antipsychotics, which usually target 5-HT_2A_ among a few other receptors, exhibit malformations^48^. In addition, mice deficient in serotonin, although seemingly normal at birth, show subsequent developmental delays^49–52^. This would suggest that serotonin and its receptors like 5-HT_2A_ may play a redundant or latent role during development; but extrinsic interventions of receptor functions by means of inhibitors/antipsychotics may still prove enormously disruptive. Since differential adhesion is likely to be critical during embryo development, malformations seen on antipsychotic treatment could conceivably be due to disruption of adhesion.

Although serotonin is widely known as a neurotransmitter, more than 90% of it is found outside the CNS^53,54^. Similarly, 5-HT_2A_ is also expressed at various non-neuronal sites such as platelets^55^, gastrointestinal tract^3^, blood vessels and vascular smooth muscle cells^56,57^, liver and spleen^2^, skin^58^, bone cells^59,60^ and cardiac fibroblasts^61^; and is attributed pre-nervous system roles as well^48^. Thus, 5-HT_2A_-mediated effects on adhesion can be expected at these sites of expression, though direct involvement is yet to be documented. The increased adhesion of HEK293 cells seen on activation of 5-HT_2A_ and the corresponding decrease seen on 5-HT_2A_ inhibition is exciting.

Besides their role as antipsychotics and antidepressants, many 5-HT_2A_ antagonists have also been widely used as anti-thrombogenic drugs^62–64^. The effects of antipsychotics are not confined to CNS and they can cause side-effects in other tissues, for e.g. eosinophilic endocarditis^65^, eosinophilic pneumonia^66^, eosinophilia^67,68^, bone fragility^69^ to name a few, and these may be directly or indirectly associated with adhesion-related processes. Interestingly, serotonin/SSRIs are even seen to promote metastasis while antipsychotics, have been reported to be remedial^70–72^.

Our study demonstrates for the first time a novel role for 5-HT_2A_ in cell–matrix adhesion and associated cytoskeletal remodelling. Since, 5-HT_2A_-mediated adhesion extends across multiple systems, from platelets to neurons, detailed studies of normal and aberrant cell adhesion in different cell systems may be also useful to understand and obviate side-effects associated with antipsychotic medications. It is essential that more studies are done to explore its significance, particularly in vivo along with a more detailed understanding of the mechanisms involved.

## Materials and methods

### Materials

5-Hydroxytryptamine/5-HT (Sigma Aldrich, H9523), 2,5-dimethoxy-4-iodoamphetamine/DOI (Sigma Aldrich, D101), 3,4-dihydroxyphenethylamine/dopamine or DA (Sigma Aldrich-H8502), chlorpromazine (Sigma, 8138), G418 and poly-d-ornithine were purchased from Sigma-Aldrich, USA. Risperidone (Tocris, 2865), olanzapine (Tocris, 4349), phorbol 12-myristate 13-acetate/PMA (Tocris, 1201) and A23187 (Tocris 52665-69-7) were purchased from Tocris, USA. Calphostin C (Cayman, 121263-19-2), CCG-1423 (Cayman 285986-88-1), SU6656, W7 (Cayman, 61714-27-0), latrunculin-A (Cayman, 76343-93-6), were purchased from Cayman Chemical, USA. Fibronectin, PrestoBlue, Phalloidin Alexa-Fluor 568 were purchased from Invitrogen, USA.

### Cell culture

HEK293 cell line was obtained from ATCC. Stable lines expressing rat 5-HT_2A_ (SB1 cells) and human 5-HT_2A_ (AB1 cells), henceforth to be called Rat_2A_SB1 and Hu_2A_AB1 cells respectively were from earlier studies^13,15^. Briefly, HEK293 cells were transfected with a neomycin-resistant plasmid containing either the rat or human 5-HT_2A_ by lipofectamine-mediated transfection, and cells stably expressing the receptors were clonally expanded and maintained in 1 mg/ml G418. The cells were routinely cultured in 10% DMEM (Invitrogen, USA) containing 10% fetal bovine serum (Invitrogen, USA), 50 U/ml penicillin and 50 µg/ml streptomycin (Invitrogen, USA) at 37 °C in 5% CO_2_.

Cells from confluent T25 flask were harvested by trypsinization, neutralized with 10% DMEM and used for experiments. For each cell adhesion experiment around 2 × 10^5^ cells in 10% DMEM (without G418) were seeded per well in 24 well dishes (Nunc, USA), either serum ECM, fibronectin or a poly-d-ornithine matrix following incubation for 24–36 h, the cell-adhesion assay was performed.

For the cell-adhesion assay, the plate was washed and the adherent cells in each well were serum-starved in 0.5 ml serum-free DMEM for 45 min at 37 °C and 5% CO_2_. Following this, the cells were treated with 5-HT_2A_ agonists (serotonin/5-HT, DOI, dopamine), antipsychotics (risperidone/chlorpromazine/olanzapine), PKC blocker (calphostin C), PKC activator (PMA), calcium ionophore (A23187), calmodulin blocker (W7), Rho signaling blocker (CCG-1423), and stress fiber disruptors (latrunculin A) as needed for 45 min unless otherwise mentioned. The solvents for the various compounds were as follows—calphostin C, PMA, W7 and latrunculin A were dissolved in ethanol, A23187 and CCG-1423 were dissolved in DMSO and the rest were dissolved in water. The final concentrations of the vehicles in the experimental medium ranged from 0.04% v/v to 0.4% v/v and a vehicle control was used in all requisite cases.

Cell-substrate interactions were disrupted by dropping the plates from a fixed height of approximately 5 cm, 30 times within a metal frame which allowed for controlled disruption. These parameters were determined previously based on the conditions required for dislodging the loosely-adherent parent cell-line. Non-adherent (floating) cells were then removed by inverting the plate and the remnants of the media were removed with a micropipette. The remaining adherent cells were trypsinized and a cell count was done using a haemocytometer (direct count) and/or using PrestoBlue dye (Invitrogen, USA) as per manufacturer’s instructions. For each treatment two samples from each well were counted in the case of haemocytometer-based method and three samples for PrestoBlue dye-based method. For PrestoBlue dye-based cell counting, 10 μl PrestoBlue dye was added to 90 μl cell suspension and incubated at 37 °C and 5% CO_2_ for 30 min in dark and the fluorescence values were used to determine the cell number (Supplementary Fig. S1).

The results were represented as the “Fraction of adherent cells”:

$$ \frac{{\left[ {\text{The number of cells remaining adherent after tapping}} \right]}}{{\left[ {{\text{The total number of cells intially present}}*} \right]}} $$

*The total number of cells initially present: while plating cells for adhesion assays, apart from the plate designated for adhesion assay, in another 24 well plate (identical matrix) at least three wells were loaded with the same number of cells and cultured under identical conditions. During the adhesion assay, the average of number of cells were determined from these three wells to serve as the most the reliable estimate of average number of cells per well (Supplementary Fig. S1).

Each experiment had each treatment in duplicate (as a minimum) and their averages were considered as one data point. Each experiment was repeated thrice for biological replicates.

### Cell attachment assay with an integrin blocker

Hu_2A_AB1 cells and HEK293 cells were harvested using an enzyme free cell dissociation buffer (Sigma, USA), re-suspended in DMEM containing dialyzed serum (which is serotonin-free) supplemented with 10 μM serotonin, and the cell suspension was diluted to 1.5 × 10^5^ cells/ml. Then 20 μM RGD peptide was added to the suspension and incubated at 37 °C and 5% CO_2_ for 60 min. Following the incubation, 6 × 10^4^ cells were plated onto fibronectin pre-coated (0.1 μg/ml, 60 min at 37 °C) wells of a 24-well dish and allowed to attach for 60 min. Finally, the media along with the unattached cells were aspirated and the attached cells were counted using PrestoBlue (Invitrogen, USA).

### Phalloidin staining

4 × 10^4^ cells were plated on 15 mm-diameter coverslip dishes pre-coated with 0.1 µg/ml fibronectin and incubated for 24–36 h for the cells to attach and spread. The dishes were washed and serum-starved in plain DMEM for 45 min. The cells were then treated with 10 µM serotonin and risperidone for 45 min. Following the treatment, the dishes were washed in phosphate buffered saline (PBS) and fixed with 4% PFA in PBS for 20 min. After fixation, the dishes were washed twice with PBS and permeabilized with 0.1% Triton for five minutes. After a further two washes with PBS, the cells were incubated with 5% BSA for 30 min to block non-specific binding, washed with PBS and stained with 50 nM Phalloidin Alexa 568 (Invitrogen, USA) in 1% BSA for 60 min. The dishes were washed twice in PBS, DABCO was added and the cells were imaged with an epifluorescence microscope.

### Image acquisition

Nikon Eclipse T2000E-PFS (Japan) attached to a Photometrix Cascade II 512 EM-CCD camera (Roper Scientific, USA) controlled by Image Pro-Plus AMS software (Media Cybernetics, USA). A 60X/NA 1.4 oil immersion objective was used. Phalloidin Alexa-Fluor 568 fluorescence was visualized through a filter procured from Chroma Technologies (Rockingham, USA): Exciter 545 nm/30, dichroic 620 nm/60, Emitter 575 nm long pass.

### Quantification of area under stress fibers

The total area under stress fiber was calculated as the cumulative area of all pixels above a threshold intensity in phalloidin-stained images using FIJI/ImageJ. Since the fluorophore only stained actin fibres and non-specific staining was very limited, the threshold was defined using controls that were not treated by ligand. Since the stress fibers had higher intensity than the background or nonspecific signals arising from other structures in the image the thresholding selected only the area occupied by stress fibers. The ratio of total area occupied by stress fibers to the total area occupied by the cells in that image was compared between the samples. The analysis consisted of ten images from each treatment. The images were all captured under identical condition, illumination, duration and gain.

### Data presentation

Some of the data was collected as a part of large, multi-parameter experiments. For the sake of clarity we have presented the results are separately in context of the substrate/drug being discussed in order to aid the comprehension of results. Due to this, some of the shared controls appear to be repeated in different figures. The details are as follows:

The data for serotonin treatment of HEK293, Hu_2A_AB1 and Rat_2A_SB1 on serum ECM in Fig. 1A is also presented in Fig. 3A. The data for serotonin treatment of Hu_2A_AB1 and Rat_2A_SB1 on fibronectin substrate in Fig. 1B is also presented in Fig. 5B. The data for DOI and DA treatment of Hu_2A_AB1 on fibronectin substrate in Fig. 1C (which shows absence and presence of DOI/DA) is also presented in Fig. 3D (which looks at DOI/DA in the absence/presence of risperidone). The data for serotonin treatment of Hu_2A_AB1 and Ra_t2A_SB1 on poly-d-ornithine substrate in Fig. 2A is also presented in Fig. 3C (where it is compared with risperidone treatment on poly-d-ornithine substrate). The data for serotonin treatment of Hu_2A_AB1 and Rat_2A_SB1 in fibronectin substrate in Fig. 3C (as a control for risperidone treatment) is also presented in Fig. 3E (as a control for olanzapine and chlorpromazine treatment) and Fig. 6B (W7 treatment control). The data for untreated control of Hu_2A_AB1 and Rat_2A_SB1 in fibronectin substrate in Fig. 1B is also presented in Supplementary Fig. S4A (as a control for calphostin C treatment).

### Statistical analysis

Data are expressed as mean ± standard deviation of the mean. The data was analysed by two tailed Student’s t test. Statistical significance was determined as *p* < 0.05.

Lilliefors goodness-of-fit test of composite normality was used to check the normality of each dataset in the case of compilation of data from the entire duration of the study. The datasets with a resultant critical value (LCRIT) values below the critical value based on the Lilliefors test table with alpha value of 0.05 is considered as a normal distribution.

## Supplementary Information

Supplementary Information.
