# Supplementary material for: Serotonin 2A (5-HT2A) receptor affects cell–matrix adhesion and the formation and maintenance of stress fibers in HEK293 cells
Source: Sci Rep. 2020 Dec 10;10:21675. doi: 10.1038/s41598-020-78595-6 (PMC7728786; doi:10.1038/s41598-020-78595-6)
Supplement: Supplementary file 1 — Supplementary Information. [file 41598_2020_78595_MOESM1_ESM.pdf]

<u>**Supplementary information**</u>

Serotonin 2A (5-HT_{2A}) receptor affects cell-matrix adhesion and the formation and maintenance of stress fibers in HEK293 cells

Joe Anand kumar John Jayakumar*, Mitradas. M. Panicker, Basudha Basu*

Supplementary Fig. S1.

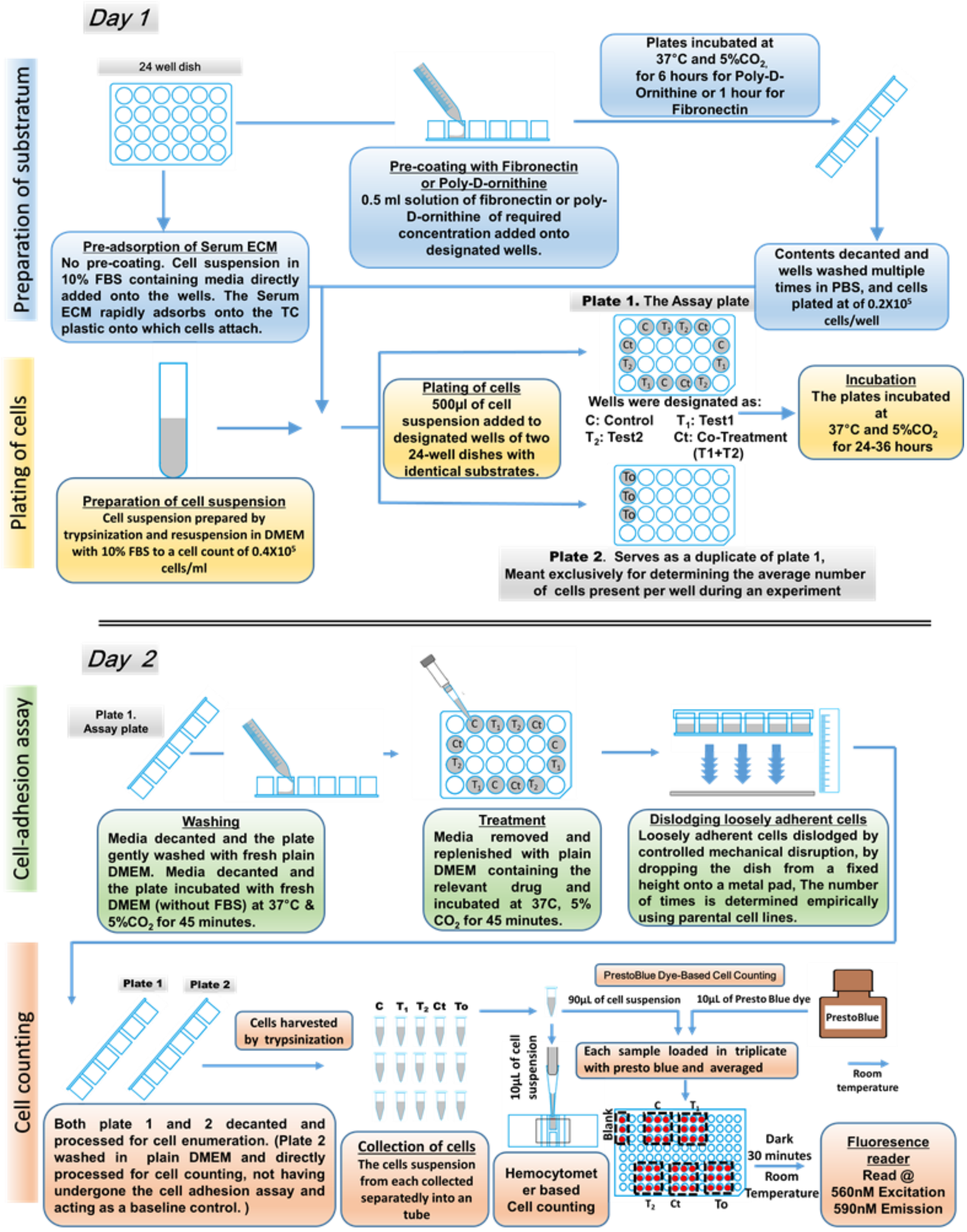

Supplementary Fig. S1. Schematic representation of the cell-adhesion assay. $0.2X10^5$ cells were grown in 10% FBS containing media, in a 24 well plate (designated as assay plate) either pre-coated with fibronectin or poly-D-ornithine, or pre-adsorbed with serum-containing media. Concurrently, the same number of cells were grown in identical growth conditions in another 24 well plate for determining the total number of cells during the experiment. Both the plates were kept in the same

incubator for 24-36 hours at 37°C and 5% CO_2, with 0.5 ml media. On the day of the experiment, the media in the assay plate was replaced with fresh DMEM (without FBS) equilibrated with 5% CO_2. This step was repeated twice to wash off FBS and the plate was placed back in the incubator at 37°C and 5% CO_2 for 45 minutes with fresh CO_2 equilibrated DMEM without FBS. Following this, the treatments were started by replacing the media in each well with CO_2-equilibrated plain DMEM containing drugs/vehicles and incubated for 37°C and 5% CO_2 for another 45 minutes (each treatment was done in a minimum of two wells). Post-treatment, the loosely adherent cells were dislodged by dropping the plate onto a metal pad from a fixed height for a certain number of times. The non-adherent cells were removed by decanting along with the media. The adherent cells in each well were trypsinized and collected individually. The cell suspensions were manually counted using a haemocytometer or using the PrestoBlue dye-based method. The total number of cells initially present was determined by directly enumerating the cells from at least three wells on the control plate. For the haemocytometer, 10µL of the cell suspension was loaded onto the Neubauer's chamber and manually counted under a microscope. For PrestoBlue dye-based method, 90µL cell suspension was mixed with 10µL Presto Blue dye, incubated at room temperature for 30 minutes, and read in a fluorescence reader at 560 nM excitation and 590 nM emission. The results were represented as a ratio of the number of adherent cells to total number of cells initially present, and was termed as 'fraction of adherent cells".

"Fraction of adherent cells"**:**

$$\frac{[\text{The number of cells remaining adherent after tapping}]}{[\text{The total number of cells intially present}]}$$

Supplementary Fig. S2.

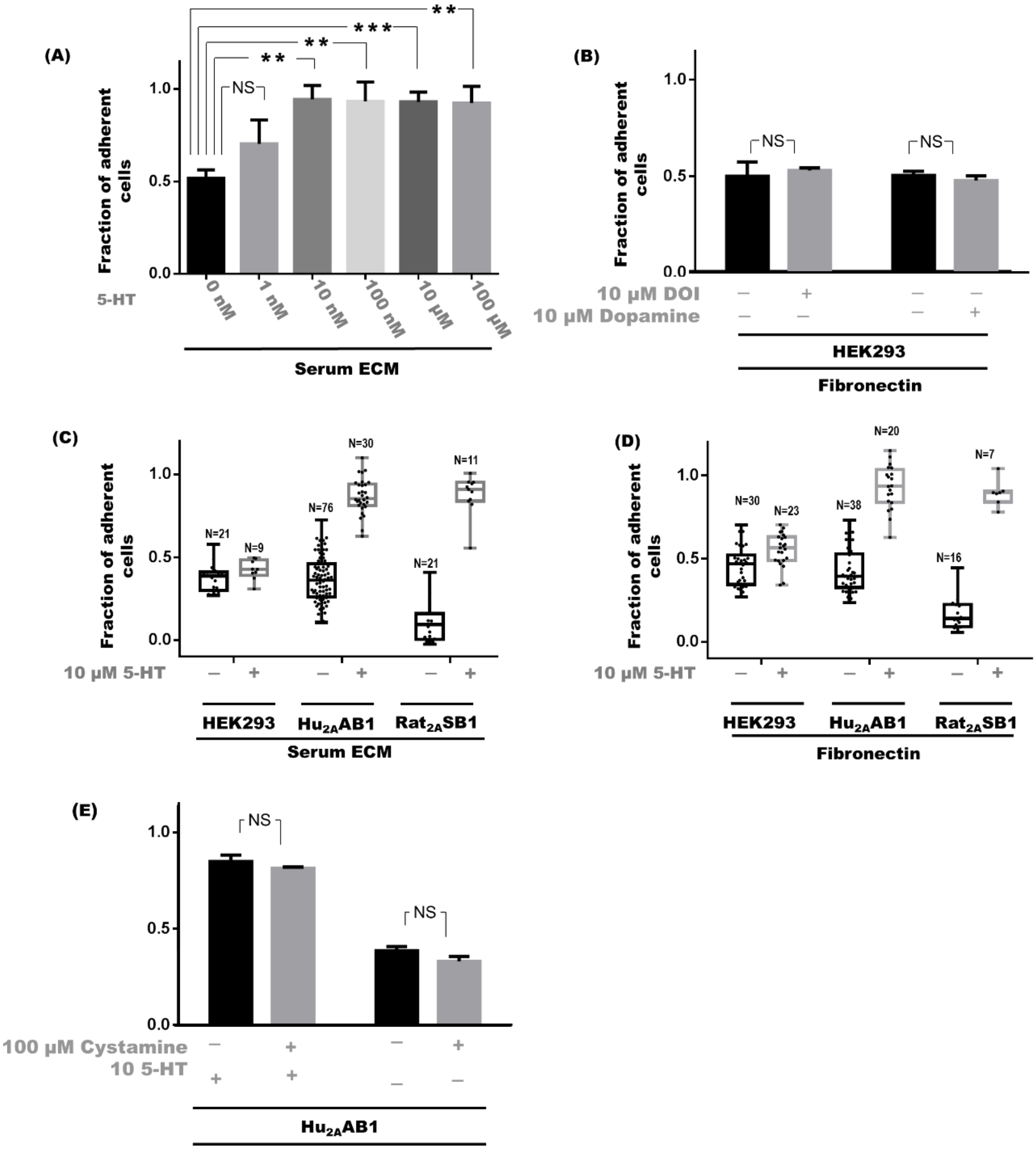

Supplementary Fig. S2. (A). Cell adhesion profile of HEK293, $Hu_{2A}AB1$ and $Rat_{2A}SB1$ cells on a serum ECM matrix compiling all the experiments done during the entire duration of the study. N=21 for HEK293, N=21 for $Rat_{2A}SB1$ cells and N=30 for $Hu_{2A}AB1$ cells.

(B). Cell adhesion profile of HEK293, $Hu_{2A}AB1$ and $Rat_{2A}SB1$ cells on a fibronectin matrix compiling all the experiments done during the entire duration of the study N=30 for HEK293 cells, N=38 for $Hu_{2A}AB1$ cells and N=16 for $Rat_{2A}SB1$ cells.

(C) Cell adhesion profile of $Hu_{2A}AB1$ cells with varying concentrations of serotonin treatment – 10 nM, 100 nM, 10 µM and 100 µM compared to untreated control on a serum-ECM substrate.

(D) Cell adhesion profile of HEK293 cells with DOI or DA.

(E) Cell adhesion profile of Hu_{2A}AB1 cells on inhibition of serotonylation with cystamine.

Error bars represent S.D. values. The data represented by the gray bars are compared against the respective black bars by two tailed students t test; p value less than 0.05 is considered significant. The corresponding p value for the asterisks are **$p<0.01$, ***$p<0.001$, NS $p>0.05$

Supplementary Fig. S3.

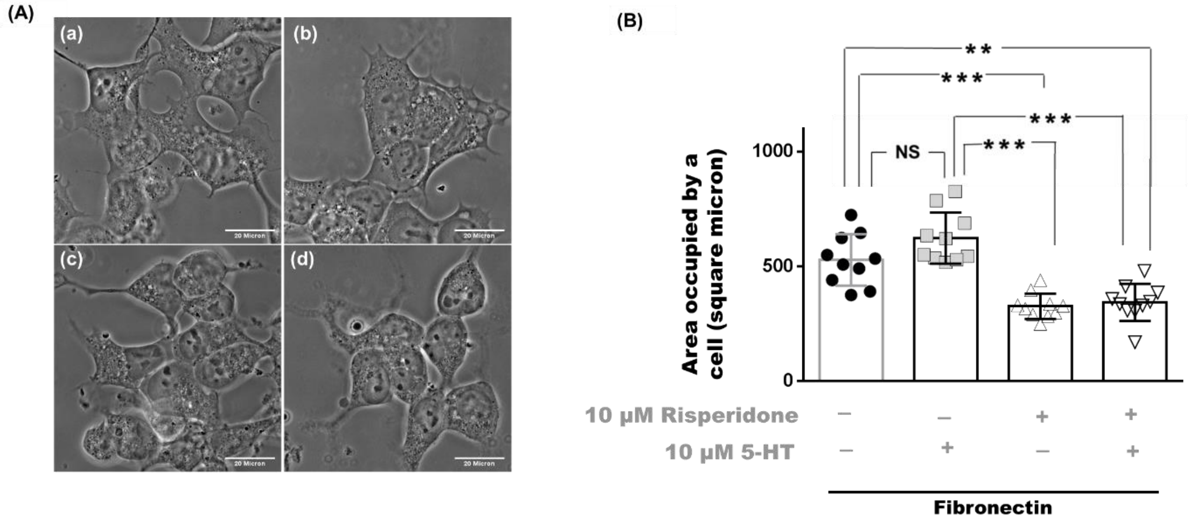

Supplementary Fig. S3. (A). Phase contrast image of untreated controls (a),and cells treated with 10 μM 5-HT (b), 10 μM risperidone (c), 10 μM risperidone + 10 μM 5-HT (d).

(B). Area occupied by $Hu_{2A}AB1$ cells under different treatments.. Control cells (black circles) measured 528.26±112.28 μm^2, and 10 μM 5-HT-treated cell (grey squares) occupied 623.21±111.12 μm^2. The area occupied by 10 μM risperidone-treated cells (upright triangles) measured 326.50±55.64 μm^2, and cells treated with 10 μM risperidone plus10 μM 5-HT (inverted triangles) occupied 343.03±80.50 μm^2.

Error bars represent S.D. values. The data represented are each compared with all other groups by two tailed students t test; p value less than 0.05 is considered significant. The corresponding p value for the asterisks are**p<0.01, ***p<0.001, NS p<0.05

Supplementary Fig. S4.

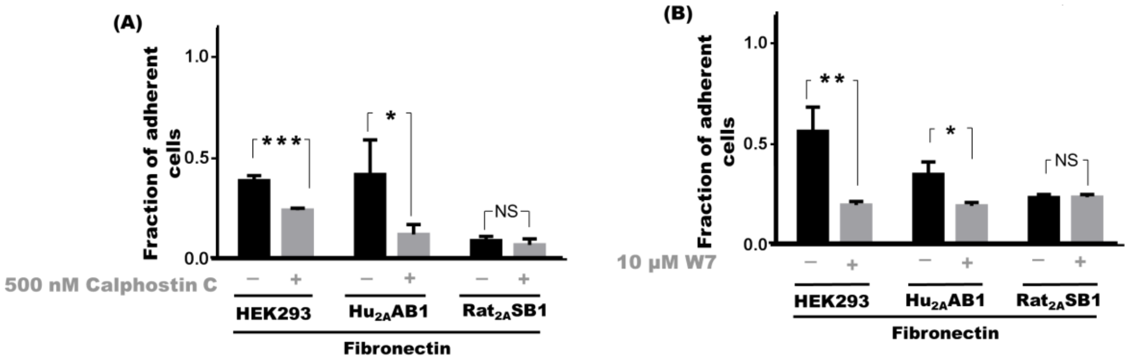

Supplementary Fig. S4. (A). Cell adhesion profile of HEK293, $Hu_{2A}AB1$ and $Rat_{2A}SB1$ cells on treatment with Calphostin C.

(B). Cell adhesion profile of HEK293, $Hu_{2A}AB1$ and $Rat_{2A}SB1$ cells on treatment with W7.

Error bars represent S.D. values. The data represented by the gray bars are compared against the respective black bars by two tailed students t test; p value less than 0.05 is considered significant. The corresponding p value for the asterisks are **$p<0.01$, *$p<0.05$, ***$p<0.001$, NS $p>0.05$

Supplementary Fig. S5.

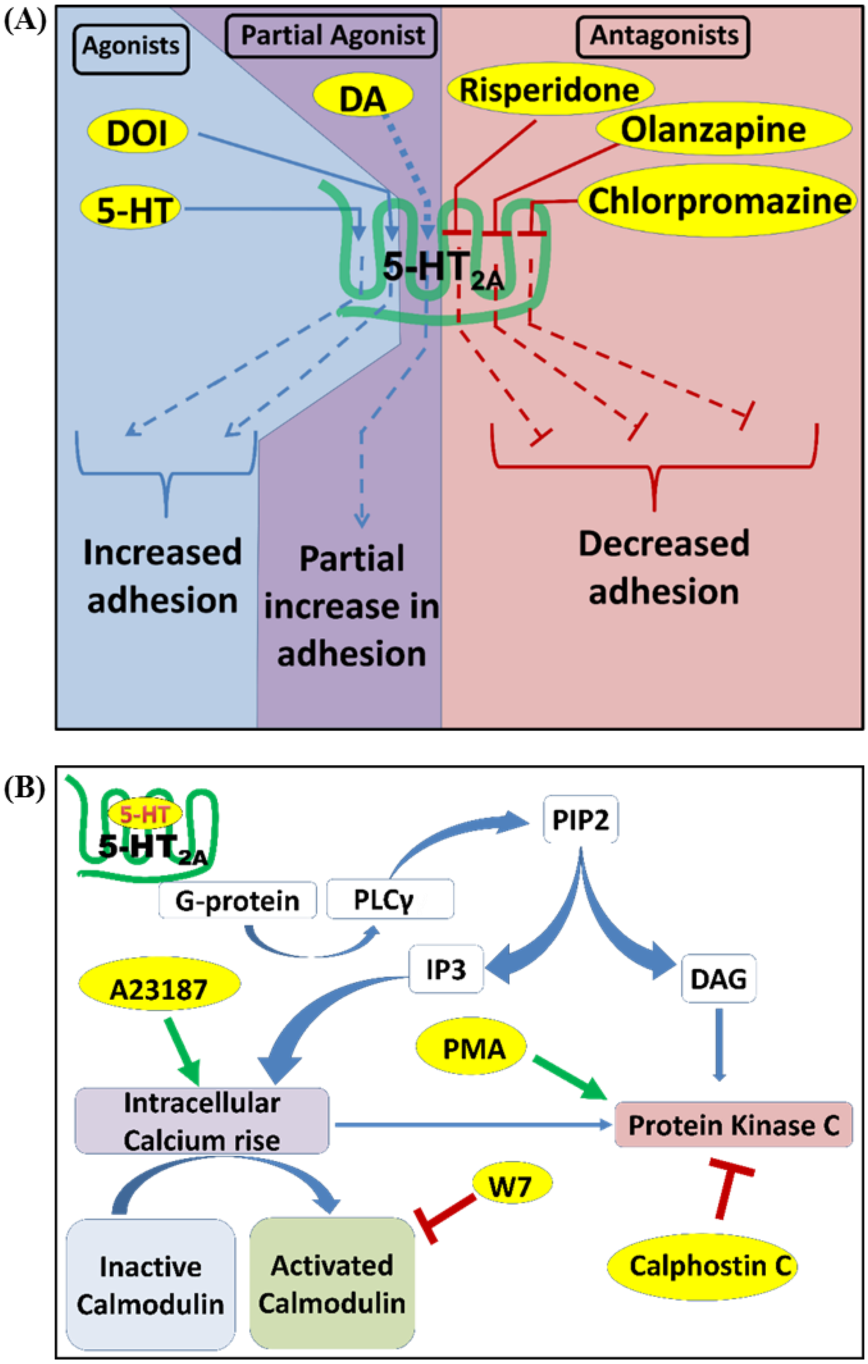

Supplementary Fig. S5. (A). Different 5-HT$_{2A}$ ligands and their effects on 5-HT$_{2A}$-mediated adhesion.

The agonists – 5-HT, DOI, DA, and antagonists –risperidone, olanzapine, chlorpromazine,

(B). The conventional 5-HT$_{2A}$ signaling components –PKC and calcium signaling and their pharmacological modulators and their effects on 5-HT$_{2A}$ -mediated adhesion. PKC is activated directly by 5-HT$_{2A}$, which can also be mimicked by PMA, and conversely, PKC can be inhibited by light-activated calphostin C. Intracellular calcium rise due to IP3 activating the calcium stores is mimicked by A23187. And the role of calmodulin is probed using W7, a blocker.
